# Unusual Cardiovascular Response to Sildenafil: Complete Heart Block in a Healthy Young Adult

**DOI:** 10.7759/cureus.78560

**Published:** 2025-02-05

**Authors:** Bodhisatwa Choudhuri, Nishant Agarwal

**Affiliations:** 1 Critical Care and Rheumatology, Parkview Super Specialty Hospital, Kolkata, IND; 2 Emergency Medicine, Charnock Hospital, Kolkata, IND

**Keywords:** autonomic modulation, av block, bradyarrhythmia, cardiovascular effects, complete heart block, myocardial ischemia, pde5 inhibitors, phosphodiesterase-5 inhibitors, sildenafil, sildenafil-induced arrhythmia

## Abstract

Sildenafil, a phosphodiesterase-5 (PDE5) inhibitor, is widely used for erectile dysfunction and pulmonary hypertension, with its cardiovascular safety profile being well documented. However, its potential to induce conduction abnormalities remains largely unexplored, both due to a lack of clinical reports and limited mechanistic studies. While tachyarrhythmias have been frequently associated with sildenafil use, bradyarrhythmias, particularly complete heart block, are an unreported complication. We present the case of a young, healthy male who developed a transient complete heart block shortly after sildenafil ingestion. Despite no prior cardiac history and normal coronary angiography, he experienced severe bradycardia unresponsive to atropine but reverted to normal sinus rhythm with isoprenaline infusion. Mechanistically, sildenafil-induced hypotension, autonomic modulation, or transient myocardial ischemia may have contributed to atrioventricular (AV) nodal suppression. Previous reports have linked sildenafil to myocardial infarction and ventricular arrhythmias, but to our knowledge, no cases of transient complete heart block have been documented. This case expands the understanding of sildenafil’s electrophysiological effects, emphasizing the need for awareness among clinicians prescribing PDE5 inhibitors. Further research is warranted to assess risk stratification for patients susceptible to sildenafil-induced conduction abnormalities.

## Introduction

Sildenafil, a selective phosphodiesterase-5 (PDE5) inhibitor, is widely used for the treatment of erectile dysfunction and pulmonary arterial hypertension. By enhancing nitric oxide-mediated vasodilation, sildenafil effectively improves penile and pulmonary vascular function. Despite its favorable cardiovascular safety profile, it has been implicated in a range of cardiovascular adverse effects, including hypotension, myocardial infarction, and various arrhythmias, predominantly tachyarrhythmias [1-4]. Its role in precipitating bradyarrhythmias, particularly high-degree atrioventricular (AV) block, remains underreported, with limited data suggesting potential associations but no conclusive evidence. Despite its vasodilatory properties, sildenafil’s effects on autonomic modulation may lead to paradoxical bradyarrhythmias in susceptible individuals.

While previous studies have predominantly explored the proarrhythmic effects of sildenafil in the context of increased sympathetic activity, its impact on AV nodal conduction and autonomic regulation remains largely unexamined [5,6]. The mechanism by which sildenafil may contribute to bradyarrhythmia is not well understood. Some studies suggest that sildenafil influences autonomic regulation of the heart, altering vagal and sympathetic balance [7]. Sildenafil may modulate baroreceptor sensitivity and autonomic tone, contributing to conduction disturbances through mechanisms beyond systemic hypotension. More specifically, sildenafil's enhancement of nitric oxide (NO) signaling and subsequent increase in cyclic guanosine monophosphate (cGMP) levels may influence vagal tone and AV nodal conduction. Elevated cGMP can increase parasympathetic activity, potentially leading to heightened AV nodal suppression and bradyarrhythmias. Given the widespread use of sildenafil and the potential for severe cardiac events, understanding its full electrophysiological impact is critical.

In this report, we present a case of transient complete heart block following sildenafil ingestion in an otherwise healthy young male, highlighting the need for further research into the electrophysiological implications of PDE5 inhibitors. It raises important questions about sildenafil’s interaction with cardiac conduction pathways and whether it could unmask latent conduction disease in certain individuals. While the possibility of unmasking latent conduction abnormalities is speculative, it underscores the need for further investigation into the potential predisposition of certain individuals to sildenafil-induced conduction disturbances.

## Case presentation

A 27-year-old previously healthy male presented to the emergency department with complaints of sudden-onset dizziness, near syncope, and profound fatigue approximately thirty minutes after ingesting sildenafil (50 mg) for the first time for recreational use. He confirmed that sildenafil was taken alone without co-ingestion of alcohol, energy drinks, or other substances. He had no known medical or surgical comorbidities, no prior history of cardiovascular disease, and was not on any medications or recreational drugs. He denied any associated chest pain, palpitations, exertional dyspnea, or similar preceding episodes.

**Figure 1:**
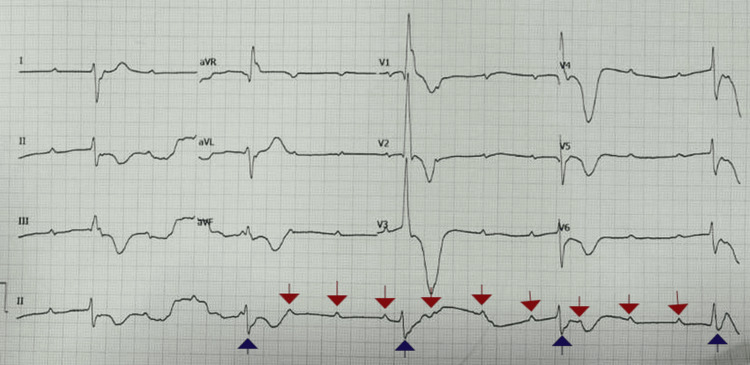
A 12-lead electrocardiogram (ECG) demonstrating complete heart block (third-degree atrioventricular block) The ECG shows a regular atrial rhythm with independent ventricular activity, indicating atrioventricular dissociation. The P waves (red downward arrows) are present but bear no consistent relationship to the QRS complexes (blue upward arrows), confirming the diagnosis of complete heart block. The ventricular escape rhythm appears junctional or low ventricular in origin, as evidenced by the narrow QRS complexes. There are no significant ST-segment elevations or depressions, suggesting an absence of acute ischemic changes.

On arrival at the emergency department, he was alert but displayed signs of lethargy and pallor. His vital signs showed significant bradycardia, with a heart rate of 28 beats per minute, and hypotension, with a blood pressure of 96/58 mmHg. His respiratory rate was 16 breaths per minute, and oxygen saturation was 98% on room air. A random blood glucose level was 145 mg/dL. Initial 12-lead electrocardiogram (ECG) (Figure 1) revealed a complete heart block with a junctional escape rhythm, demonstrating AV dissociation with regular but independent P waves and a slow ventricular response. The QRS complexes were narrow, suggesting a junctional escape focus, and there were no ischemic changes or ST elevations. Arterial blood gas analysis was unremarkable (pH 7.41, partial pressure of oxygen (PaO2) 95 mmHg, partial pressure of carbon dioxide (PaCO2) 40 mmHg, bicarbonate (HCO3-) 24.5 mEq/L, base excess 1) except for a mild lactate elevation of 1.8 mmol/L, likely secondary to transient hypotension.

**Figure 2:**
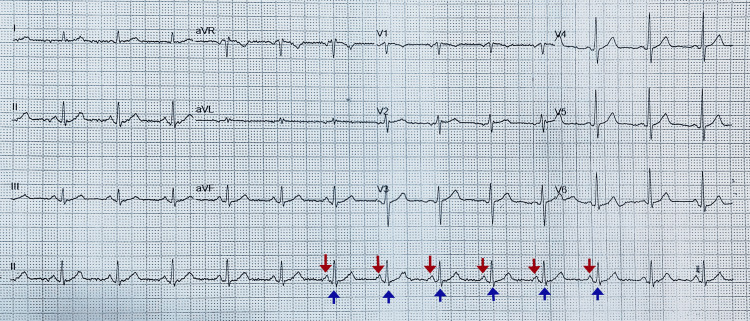
A 12-lead electrocardiogram (ECG) demonstrating normal sinus rhythm following the resolution of complete heart block The ECG shows a regular rhythm with appropriately timed P waves (red downward arrows) preceding each QRS complex (blue upward arrows), confirming intact atrioventricular conduction. The QRS complexes are narrow, indicating normal ventricular depolarization. There are no ST-segment elevations or depressions and no evidence of conduction abnormalities or ischemic changes.

The patient was stabilized using the airway, breathing, circulation, disability, and exposure (ABCDE) approach, with intravenous access and continuous cardiac monitoring. A one-liter bolus of normal saline was administered, following which his blood pressure improved to 124/78 mmHg. Despite receiving three sequential doses of intravenous atropine (1 mg each) at intervals of five minutes, there was no improvement in heart rate. Given the persistence of complete heart block, isoprenaline infusion was initiated at 5 mcg/min while preparations were being made for transvenous pacing. The infusion rate was increased to 10 mcg/min after ten minutes due to persistent bradycardia. Approximately twenty minutes after initiation of isoprenaline, the patient spontaneously reverted to normal sinus rhythm, and transvenous pacing was ultimately not required. Post-stabilization, his heart rate increased to 78 beats per minute, his blood pressure remained stable at 132/80 mmHg, and his oxygen saturation was 99% on room air. Repeat ECG (Figure 2) confirmed normal sinus rhythm without residual conduction abnormalities.

**Figure 3:**
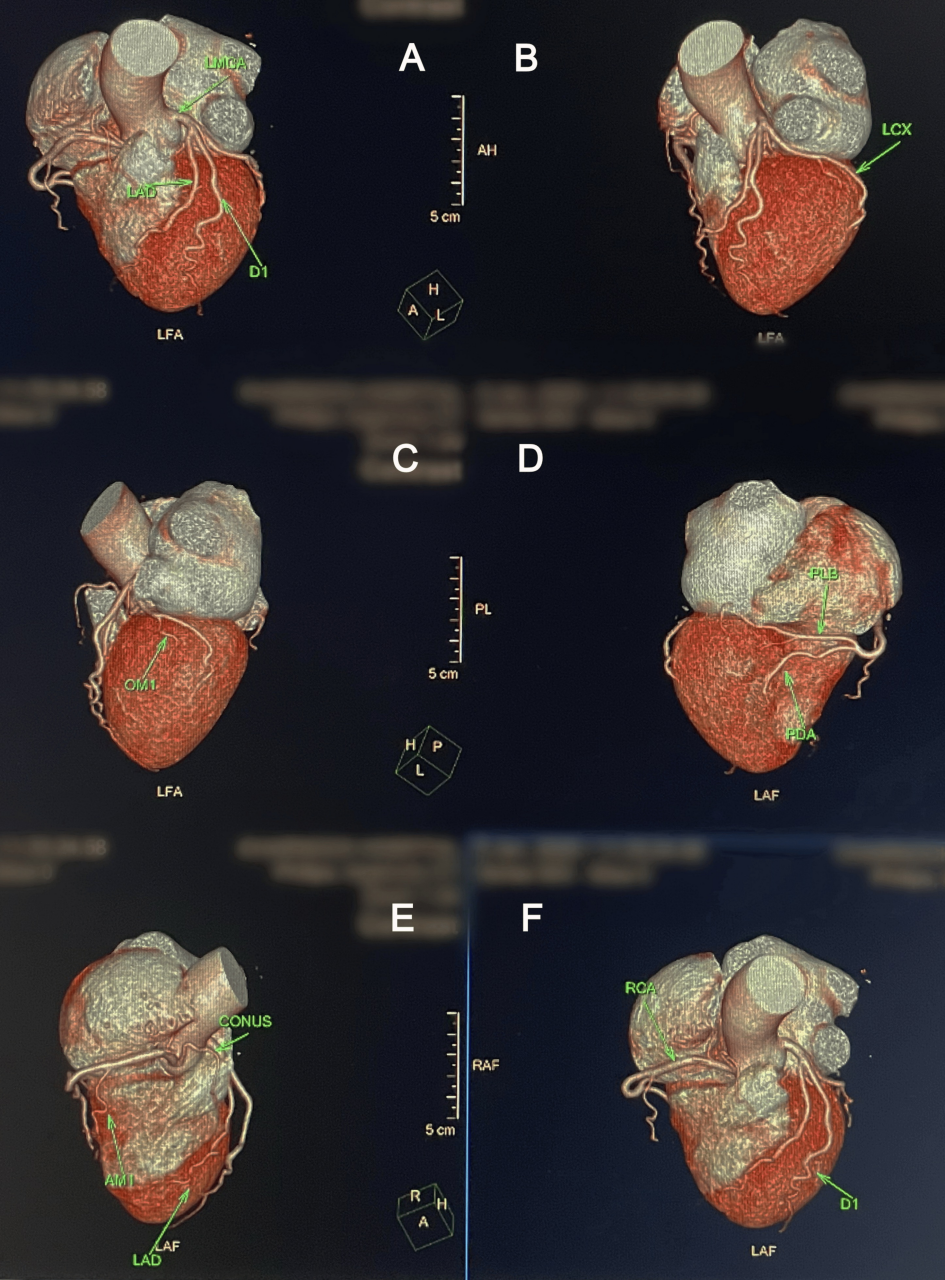
Multiplanar 3D reconstructed CT coronary angiography images in different orientations showing normal coronary anatomy A: anterior view displaying the left main coronary artery (LMCA), left anterior descending artery (LAD), and diagonal (D1) branches; B: left lateral view highlighting the left circumflex (LCX) artery; C: left oblique view showing the obtuse marginal (OM1) branch of the LCX; D: inferior view demonstrating the posterior descending artery (PDA) and posterolateral branch (PLB) of the right coronary system; E: anterior-inferior view identifying the conus artery, LAD, and acute marginal (AMI) branch; F: right anterior oblique view illustrating the right coronary artery (RCA) and D1 branch. This imaging confirms the absence of coronary artery disease or structural cardiovascular pathology in the patient. All patient identification details, including name, hospital name, date, and time, have been blurred to maintain confidentiality.

The patient was admitted to the intensive care unit for close monitoring. Serial ECGs throughout hospitalization remained normal, and there was no recurrence of bradyarrhythmia. Transthoracic echocardiography showed normal biventricular function with no structural heart disease or valvular abnormalities. Chest X-ray and high-resolution computed tomography (HRCT) of the thorax were normal, with no evidence of structural lung or mediastinal abnormalities. A 24-hour Holter monitor did not reveal any further conduction disturbances. Given the unusual presentation, a computed tomography coronary angiography (CT-CAG) (Figure 3) was performed, which showed normal coronary arteries, effectively ruling out ischemic heart disease as a cause of conduction abnormalities. The clinical relevance of the patient’s normal echocardiogram and CT coronary angiography lies in the exclusion of structural heart disease and ischemia, supporting a functional rather than anatomical cause for the conduction disturbance.

Serial cardiac biomarkers, including troponin, remained within normal limits (Table 1). Viral myocarditis was not suspected as there were no clinical signs or symptoms suggestive of such, and inflammatory markers remained within normal limits.

**Table 1:** Laboratory investigations Baseline-on presentation to ED (day zero), and the day of discharge (day three) ED: emergency department; CRP: C-reactive protein; WBC: white blood corpuscles; AST: aspartate aminotransferase; ALT: alanine aminotransferase; NT-proBNP: N-terminal pro-B-type natriuretic peptide

Tests	Results (on ED presentation)	Results (on the day of discharge)	Reference ranges
Hemoglobin	15.3 g/dl	15.1 g/dl	14-18 g/dl
WBC count	7840 cells/mm^3^	9760 cells/mm^3^	4500-11000 cells/mm^3^
Platelets	165000 cells/mm^3^	230000 cells/mm^3^	150000-350000 cells/mm^3^
CRP	1.6 mg/l	1.2 mg/l	<3 mg/l
Urea	17.5 mg/dl	15.6 mg/dl	6-24 mg/dl
Creatinine	1.02 mg/dl	0.86 mg/dl	0.6-1.2 mg/dl
Sodium	142 mEq/l	141 mEq/l	136-142 mEq/l
Potassium	4.1 mEq/l	3.9 mEq/l	3.5-5 mEq/l
Calcium	9.18 mg/dl	9.32 mg/dl	8.2-10.2 mg/dl
Magnesium	1.75 mEq/l	1.86 mEq/l	1.4-2.1 mEq/l
Total bilirubin	0.2 mg/dl	0.2 mg/dl	0.1-0.3 mg/dl
Albumin	4.5 g/dl	4.4 g/dl	3.5-5 g/dl
AST	37 U/l	34 U/l	10-40 U/l
ALT	39 U/l	40 U/l	10-55 U/l
Procalcitonin	<0.05 ng/ml	-	<0.05 ng/ml
Troponin-I	<0.04 ng/ml	<0.04 ng/ml	<0.04 ng/ml
NT-proBNP	47 pg/ml	19 pg/ml	<125 pg/ml
D-dimer	134 ng/ml	148 ng/ml	<500 ng/ml
Urine pus cells	1-2/hpf	-	0-2/hpf

Sildenafil was identified as the most probable causative factor after excluding other potential etiologies. Over the next 48 hours, the patient remained clinically stable, and repeat laboratory investigations were entirely normal. He was discharged in a stable condition with a follow-up plan and advised to avoid PDE5 inhibitors in the future.

As part of further outpatient evaluation, an electrophysiology study was performed, which was also entirely normal, ruling out intrinsic conduction system disease. A cardiac MRI could have provided further insights into autonomic function and potential long-term risks for conduction disturbances; however, it could not be performed as the patient was claustrophobic. Multiple follow-ups over the next six months showed no recurrence of symptoms or conduction abnormalities. The patient remained asymptomatic and did not experience any further syncopal or bradyarrhythmic episodes. 

## Discussion

Sildenafil exerts its pharmacological action by selectively inhibiting phosphodiesterase-5 (PDE5), an enzyme responsible for the breakdown of cyclic guanosine monophosphate (cGMP) in vascular smooth muscle. PDE5 is highly expressed in the corpus cavernosum and pulmonary vasculature, which explains its primary therapeutic uses in erectile dysfunction and pulmonary arterial hypertension. By increasing intracellular cGMP levels, sildenafil promotes relaxation of smooth muscle cells, leading to vasodilation in the pulmonary and systemic circulation. Although it is primarily used for erectile dysfunction and pulmonary hypertension, its cardiovascular effects extend beyond its primary indications [1]. While vasodilation is generally beneficial, sildenafil can induce systemic hypotension, which, in susceptible individuals, may trigger adverse cardiovascular effects such as myocardial infarction and arrhythmias [2,8].

The cardiovascular adverse effects of sildenafil have been widely documented, with tachyarrhythmias, including ventricular tachycardia and atrial fibrillation, being the most commonly reported complications [5,6]. The estimated incidence rates of these arrhythmias remain low but are clinically significant due to their potential severity. The occurrence of bradyarrhythmias, particularly complete heart block, is exceptionally rare, making this case noteworthy. While previous studies have associated sildenafil with myocardial infarction [9,10], even in patients without coronary artery disease [3,4], there is a significant lack of evidence linking sildenafil to AV conduction disturbances. To our knowledge, this case represents the first documented occurrence of complete heart block following sildenafil ingestion in an otherwise healthy young male. It is possible that this represents an idiosyncratic reaction unique to this individual.

The mechanism underlying sildenafil-induced bradyarrhythmia remains speculative. One potential hypothesis is an exaggerated vagal response to sildenafil, resulting in heightened parasympathetic activity and subsequent AV nodal suppression. Studies have shown that sildenafil can modulate autonomic function, increasing parasympathetic tone in some individuals [7]. Additionally, sildenafil has been reported to alter baroreflex sensitivity, leading to paradoxical bradycardia despite systemic hypotension [7]. The modulation of cGMP pathways may further influence AV node refractoriness, contributing to transient conduction block. Another possible explanation involves sildenafil-induced systemic hypotension leading to transient myocardial ischemia, affecting the conduction system despite the absence of significant coronary artery disease on angiography. The findings of Andishmand et al. [11] suggest that sildenafil plays a role in the coronary slow flow phenomenon, reinforcing its significant vasodilatory effects, which could contribute to hemodynamic instability and arrhythmias. The absence of structural heart disease, a normal electrophysiology study, and the transient nature of the conduction disturbance further suggest a functional rather than an anatomical etiology.

Frantzen et al. [12] highlighted an increased cardiovascular risk among sildenafil users seeking treatment for erectile dysfunction, emphasizing the need for preemptive cardiovascular screening in these patients. Additionally, Wysowski et al. [13] noted a rise in sudden cardiac deaths among sildenafil users, suggesting the possibility of unrecognized electrophysiological effects, which may extend to bradyarrhythmias. The study by Mittleman et al. [14] investigated cardiovascular outcomes in sildenafil users, finding no significant increase in cardiovascular mortality; however, the lack of focus on bradyarrhythmias suggests that such cases may be underreported rather than absent.

A comparison with other vasodilators and PDE inhibitors reveals that while nitrates and calcium channel blockers have been implicated in bradyarrhythmias due to excessive vasodilation and direct AV nodal suppression, respectively, similar reports with tadalafil and vardenafil are undocumented. This raises the question of whether sildenafil exerts unique effects on cardiac conduction beyond those observed with other PDE5 inhibitors.

The choice of isoprenaline over dopamine in this case was guided by its direct beta-adrenergic action, which enhances AV nodal conduction and increases heart rate. Dopamine, although useful for hypotension, has a less predictable impact on AV nodal function, making it a secondary option in this context [15].

This case underscores the rare but significant risk of sildenafil-induced complete heart block, a phenomenon not previously well documented. While sildenafil is generally considered safe, its cardiovascular effects extend beyond the widely reported tachyarrhythmias to include conduction disturbances. Clinicians should maintain a high index of suspicion for sildenafil-induced bradyarrhythmias in patients presenting with unexplained syncope or profound bradycardia. The initial workup should prioritize a 12-lead ECG to identify conduction abnormalities, echocardiography to assess structural heart disease, and consideration of autonomic function testing if symptoms persist or recur. Given the increasing use of sildenafil and its potential to induce conduction disturbances, further investigation is necessary to determine the extent of its impact on cardiac electrophysiology. A prospective study evaluating sildenafil’s effects on PR interval prolongation in healthy adults could provide valuable insights into its conduction-related effects. Such a study should include baseline ECG measurements, stress testing with a sildenafil challenge, and continuous monitoring to detect transient conduction disturbances.

Further research should aim to identify potential risk factors, including genetic predispositions, autonomic dysfunction, and baseline ECG abnormalities such as first-degree AV block. A more structured approach to identifying at-risk individuals could involve assessing factors such as age, underlying autonomic dysfunction, electrolyte imbalances, and pre-existing conduction abnormalities. Clinicians should carefully evaluate these factors before prescribing sildenafil and reconsider its use in patients with known predisposing conditions to prevent similar adverse events.

## Conclusions

This case highlights a rare but serious adverse effect of sildenafil-induced complete heart block in a healthy young adult, emphasizing the complexity of sildenafil's cardiovascular impact. While generally considered safe, sildenafil can provoke both tachyarrhythmias and bradyarrhythmias, likely due to its influence on autonomic function and myocardial perfusion. Clinicians should maintain a high index of suspicion for sildenafil-induced bradyarrhythmias, particularly in patients presenting with unexplained syncope or severe bradycardia. Given its widespread use, further research is needed to explore sildenafil’s electrophysiological effects, especially in individuals with latent conduction abnormalities, and to assess whether regulatory guidelines need re-evaluation for certain at-risk populations.

The case also underscores the importance of preemptive cardiovascular evaluation in individuals with subtle signs of autonomic dysfunction or a family history of conduction disorders, even among those using sildenafil recreationally. Despite normal laboratory results, biomarkers, and imaging studies in this patient, the transient complete heart block observed points to the unpredictability of sildenafil’s effects, possibly due to autonomic modulation, hypotension, or idiosyncratic reactions. This case calls for heightened clinician vigilance, cautious use of PDE5 inhibitors, and comprehensive patient education regarding potential cardiovascular risks, even in the absence of pre-existing heart conditions.
